# Inter- and Intra-Subunit Butanol/Isoflurane Sites of Action in the Human Glycine Receptor

**DOI:** 10.3389/fnmol.2016.00045

**Published:** 2016-06-14

**Authors:** Mandy L. McCracken, Giorgio Gorini, Lindsay M. McCracken, R. Dayne Mayfield, R. Adron Harris, James R. Trudell

**Affiliations:** ^1^Waggoner Center for Alcohol and Addiction Research, The University of Texas at AustinAustin, TX, USA; ^2^Integrative Neuroscience Research Branch, Neurobiology of Addiction Section, National Institute on Drug Abuse, National Institutes of HealthBaltimore, MD, USA; ^3^Department of Anesthesia and Beckman Program for Molecular and Genetic Medicine, Stanford School of MedicineStanford, CA, USA

**Keywords:** GLRA1, alcohol, anesthetic, *Xenopus* oocytes, GluCl model, mass spectrometry, immunoblotting, proteomics

## Abstract

Glycine receptors (GlyRs) mediate inhibitory neurotransmission and are targets for alcohols and anesthetics in brain. GlyR transmembrane (TM) domains contain critical residues for alcohol/anesthetic action: amino acid A288 in TM3 forms crosslinks with TM1 (I229) in the adjacent subunit as well as TM2 (S267) and TM4 (Y406, W407, I409, Y410) in the same subunit. We hypothesized that these residues may participate in intra-subunit and inter-subunit sites of alcohol/anesthetic action. The following double and triple mutants of *GLRA1* cDNA (encoding human glycine receptor alpha 1 subunit) were injected into *Xenopus laevis* oocytes: I229C/A288C, I229C/A288C/C290S, A288C/Y406C, A288C/W407C, A288C/I409C, and A288C/Y410C along with the corresponding single mutants and wild-type *GLRA1*. Butanol (22 mM) or isoflurane (0.6 mM) potentiation of GlyR-mediated currents before and after application of the cysteine crosslinking agent HgCl_2_ (10 μM) was measured using two-electrode voltage clamp electrophysiology. Crosslinking nearly abolished butanol and isoflurane potentiation in the I229C/A288C and I229C/A288C/C290S mutants but had no effect in single mutants or wild-type. Crosslinking also inhibited butanol and isoflurane potentiation in the TM3-4 mutants (A288C/Y406C, A288C/W407C, A288C/I409C, A288C/Y410C) with no effect in single mutants or wild-type. We extracted proteins from oocytes expressing I229C/288C, A288C/Y410C, or wild-type GlyRs, used mass spectrometry to verify their expression and possible inter-subunit dimerization, plus immunoblotting to investigate the biochemical features of proposed crosslinks. Wild-type GlyR subunits measured about 50 kDa; after crosslinking, the dimeric/monomeric 100:50 kDa band ratio was significantly increased in I229C/288C but not A288C/Y410C mutants or wild-type, providing support for TM1-3 inter-subunit and TM3-4 intra-subunit crosslinking. A GlyR homology model based on the GluCl template provides further evidence for a multi-site model for alcohol/anesthetic interaction with human GLRA1.

## Introduction

Glycine receptors (GlyRs) are members of the Cys-loop family of pentameric ligand-gated ion channels, which also include the nicotinic acetylcholine receptor (nAChR), serotonin-3 receptor (5-HT_3_R), and γ-aminobutyric acid type A receptor (GABA_A_R). GlyRs mediate the majority of inhibitory neurotransmission in the brain stem and spinal cord (Legendre, 2001) and are also expressed in the amygdala, cortex, cerebellum, hippocampus, striatum, nucleus accumbens, and the ventral tegmental area (van den Pol and Gorcs, 1988; Takahashi et al., 1992; Fatima-Shad and Barry, 1993; Molander and Soderpalm, 2005; Baer et al., 2009; Jonsson et al., 2012). Functional GlyRs are composed of five subunits positioned around a central chloride ion channel. Each subunit consists of an extracellular N-terminal domain, a transmembrane (TM) domain with four alpha helical segments (TM1, TM2, TM3, and TM4), an intracellular loop between TM3 and TM4, and an extracellular C-terminal segment. Four GlyR α subunits (1-4) and one β subunit have been identified (Grenningloh et al., 1990; Harvey et al., 2000, 2004), which can assemble to form either homomeric α or heteromeric αβ receptors (Lynch, 2004).

Alcohols and volatile anesthetics potentiate GlyR function (Harrison et al., 1993; Mascia et al., 1996) and likely share common sites of action (Mihic et al., 1997; Beckstead et al., 2001). Critical amino acid residues for alcohol/anesthetic enhancement of the human alpha 1 subunit have been identified in TM1 (I229), TM2 (S267), and TM3 (A288) (Mihic et al., 1997; Mascia et al., 2000; Lobo et al., 2004a). Less is known about TM4 residues (Bertaccini et al., 2010), but together with TM1-3 residues, they may also participate in water-filled, alcohol/anesthetic sites of action within the TM domain (Mascia et al., 2000; Jenkins et al., 2001; Trudell and Harris, 2004; Lobo et al., 2004a, 2006). For example, A288 in TM3 forms crosslinks with the critical residues for alcohol/anesthetic action in TM1 (I229) (Lobo et al., 2008), TM2 (S267) (Lobo et al., 2004b), and TM4 (Y406, W407, I409, and Y410) (McCracken et al., 2010). In addition, sites for alcohol have been identified near the ion pore in nAChRs (Borghese et al., 2003) and GABA_*A*_Rs (Borghese et al., 2016).

Crosslinking of A288 in TM3 with Y406, W407, I409, or Y410 in TM4 supports the assignment of these TM4 residues as intra-subunit facing (Bertaccini et al., 2010; McCracken et al., 2010). X-ray structures of anesthetics bound to the bacterial homolog GLIC (*Gloeobacter violaceus* ligand-gated ion channel) and photolabeling of GABA_A_Rs further support action at an intra-subunit cavity (Nury et al., 2010; Yip et al., 2013). However, other research proposed an inter-subunit drug pocket (Bali et al., 2009), suggesting that the homologous residue to A288 in TM3 of the GABA_A_R is oriented toward the subunit interface, such that it is able to form crosslinks with TM1 residues of an adjacent subunit. Photoaffinity labeling indicated that multiple classes of general anesthetics act at an inter-subunit site (Stewart et al., 2008; Zhong et al., 2008; Li et al., 2010; Chiara et al., 2013). Crystallography, paired with functional studies in GLIC, was used to compare the receptors in the presence and absence of ethanol and bromoethanol and provided support for inter-subunit binding cavities (Sauguet et al., 2013). A multi-site hypothesis has also emerged suggesting that alcohols and anesthetics act at both intra- and inter-subunit GlyR sites. This hypothesis resulted largely from mutagenesis studies of the bacterial GLIC and invertebrate GluCl (glutamate-gated chloride channel) homologs, molecular simulations, and homology modeling (Howard et al., 2011; Murail et al., 2012; Yoluk et al., 2013). Crosslinking studies of the mammalian GABA_A_R provided further evidence for this model (Borghese et al., 2014). Recent electron cryo-microscopy structures of the zebrafish GlyR in the glycine-, glycine/ivermectin-, and strychnine-bound states (Du et al., 2015) have also provided strong support for our homology models.

In the present study, we used cysteine mutagenesis in relevant TM locations and tested the proximity of cysteine pairs using crosslinking agents, electrophysiology, immunoblotting, mass spectrometry (MS), and computer modeling. We determined if crosslinking of A288 in TM3 with critical residues in TM1 or TM4 alters alcohol or volatile anesthetic potentiation of the human GLRA1 subunit and if drug binding sites likely exist within a subunit or between adjacent subunits. Evidence for intra-subunit vs. inter-subunit sites of drug action was inferred using electrophysiology, biochemistry, proteomics, and structural modeling, although these approaches do not specifically distinguish between active vs. allosteric sites.

## Materials and methods

### Materials

Adult female *Xenopus laevis* frogs were purchased from Nasco (Fort Atkinson, WI). Glycine was purchased from Bio-Rad Laboratories (Hercules, CA), isoflurane was from Marsam Pharmaceuticals, Inc. (Cherry Hill, NJ), and all other chemicals and buffer ingredients were from Sigma-Aldrich (St. Louis, MO).

### Site-directed mutagenesis

Point mutations in *GLRA1* cDNA (subcloned in the pBKCMV N/B-200 vector) were made using a QuickChange site-directed mutagenesis kit (Stratagene, La Jolla, CA). Single mutants (A288C, I229C, Y406C, W407C, I409C, Y410C), double mutants (I229C/A288C, I229C/C290S, A288C/Y406C, A288C/W407C, A288C/I409C, A288C/Y410C), and a triple mutant (I229C/A288C/C290S) were constructed. All point mutations were verified by DNA sequencing in the core facility at The University of Texas at Austin.

### Oocyte isolation and cDNA injection

Adult *Xenopus* females were housed in a state-of-the-art frog facility designed and installed by Aquatic Habitats located in the Animal Resources Center at The University of Texas at Austin. Frogs were anesthetized in a tricaine immersion bath and portions of ovary were surgically extracted in accordance with an approved Institutional Animal Care and Use Committee protocol (AUP-2015-00205). Mature oocytes were manually isolated, treated in 0.5 mg/ml collagenase (type IA), and subsequently injected with 30 nl of nuclease-free water containing *GLRA1* (1.5 ng/30 nl) wild-type or mutant cDNA into the nucleus. Injected oocytes were incubated at 15°C in sterile modified Barth's solution (MBS containing: 88 mM NaCl, 1 mM KCl, 2.4 mM NaHCO_3_, 19 mM HEPES, 0.82 mM MgSO_4_, 0.33 mM Ca(NO_3_)_2_, 0.91 mM CaCl_2_, 10,000 units/L penicillin, 50 mg/L gentamicin, 90 mg/L theophylline, 220 mg/L sodium pyruvate, pH 7.5) for 1-7 days.

### Electrophysiological recording

Oocytes were placed in a 100-μl bath with the animal poles facing upwards and impaled with two high-resistance (0.5–10M) glass electrodes filled with 3M KCl. There were two ground wires (on either side of the oocyte) in the bath and both were plugged into the amplifier. In addition, two electrodes (one monitors voltage, the other injects current in voltage clamp mode) were inserted into the oocyte. Cells were voltage clamped at −70 mV using an OC-725C oocyte clamp (Warner Instruments, Hamden, CT) and perfused with MBS at a rate of 2 ml/min using a Masterflex USA peristaltic pump (Cole Parmer Instrument Co., Vernon Hills, IL) through 18-gauge polyethylene tubing. All drug solutions were freshly prepared in MBS. Currents were acquired using a Powerlab 4/30 digitizer with LabChart version 7 software (ADInstruments, Bella Vista, NSW, Australia). Peak currents were measured and used in data analysis. Currents observed in the presence of glycine plus modulators were compared with currents generated by glycine alone.

#### Alcohol and volatile anesthetic responses before and after crosslinking

The glycine EC_5−10_ (the concentration that produced 5–10% of the maximal response) was determined for each oocyte after a 15-s application of the maximal glycine concentration (100 mM), and this served as the test concentration. Individual oocytes and receptors vary in glycine sensitivity, and thus we indicate glycine EC_5−10_ rather than specifying the different glycine concentrations that were used in each set of experiments. We studied glycine EC_5−10_ because potentiation by alcohols and anesthetics is best observed at these concentrations. Typical EC_50_ values for wild-type and mutant receptors are listed in McCracken et al. (2010). The test concentration was applied for 30 s followed by a 5-min washout. Two consecutive glycine applications were applied to ensure that responses were stable. Butanol or isoflurane was pre-applied for 1 min and then co-applied with glycine for 30 s followed by a 10-min washout. The test glycine concentration was reapplied, and the percent potentiation of the glycine-induced current by butanol or isoflurane was calculated for each oocyte. HgCl_2_ (10 μM) was applied for 1 minute (in the absence of glycine for the A288C/W407C and A288C/Y410C mutants and in the presence of 100 mM glycine for the A288C/Y406C, A288C/I409C, I229C/A288C, and I229C/A288C/C290S mutants) followed by a 15-min washout (McCracken et al., 2010). The maximal glycine concentration was then reapplied and the test concentration (EC_5−10_) recalculated. The potentiation of glycine-induced current by the alcohol or anesthetic was measured again as described above. The concentrations of butanol (22 mM) or isoflurane (0.6 mM) used correspond to approximately two times the minimal alveolar concentration needed to suppress movement to a painful stimulus.

#### Oxidation with H_2_O_2_ and reduction with DTT

In addition to the chemical crosslinking agent HgCl_2_ that was used to form S-Hg-S bonds, we replicated those results using oxidation of the thiol groups of the double mutants to form S-S bonds. The maximal glycine concentration (100 mM) was applied for 15–20 s followed by a 15-min washout. This concentration was reapplied and the second response served at the test response. H_2_O_2_ (0.5%) was then applied in the presence of 100 mM glycine for 1 min followed by a 15-min washout. The oocytes were unclamped during application and washout of H_2_O_2_, but remained impaled by the electrodes as previously described (Lobo et al., 2008; McCracken et al., 2010). After re-clamping to −70 mV, 100 mM glycine was applied and the responses were compared to the glycine test response before H_2_O_2_ application. Glycine (100 mM) was applied a second time and served as the new test response. Then crosslinks were reduced by applying dithiothreitol (DTT, 10 mM) for 3 min to unclamped oocytes followed by a 15-min washout period before being re-clamped. Glycine (100 mM) was reapplied and the responses were compared to the glycine test response following H_2_O_2_ application.

#### Data analysis

Three to six oocytes were used per experimental condition (see figure legends). For each receptor tested with HgCl_2_, repeated measures *t*-tests were used to detect differences before and after crosslinking (i.e., pre- vs. post-HgCl_2_ or pre- vs. post- H_2_O_2_). For the crosslinking experiments using the oxidizing agent H_2_O_2_ and reducing agent DTT, a one-way ANOVA with Tukey's *post-hoc* test was used to examine significant differences between conditions (pre-H_2_O_2_ vs. post-H_2_O_2_ vs. post-DTT). GraphPad Prism software (La Jolla, CA) was used for all analyses, and the threshold for statistical significance was set at *P* < 0.05.

### Protein extraction

Oocytes were manually isolated and injected with 1.5 ng/nl cDNA as described above and then incubated in MBS for 5–7 days at 13°C. Groups of 25 oocytes were pooled for each condition. Oocytes in the crosslinked group were treated with 0.5% H_2_O_2_ by bath perfusion according to the protocol described earlier for HgCl_2_ application (also see McCracken et al., 2010). Following a 15-minute washout, oocytes were homogenized in 1 ml of Wash Buffer (0.1 M EDTA, pH 7.5; 0.1 M EGTA, pH 7.5; 2M NaCl; 0.1M NaH_2_PO_4_, pH 7.5) with protease inhibitors (5 mM benzamidine and 15 mM iodoacetamide), and then centrifuged at 4°C for 30 min at 13,500 rpm. The supernatant was removed, and 250 μl of Extraction Buffer (Wash Buffer + 2% Triton + 5 mM benzamidine + 15 mM iodoacetamide) was added. The pellet was resuspended, rotated at 4°C for 2 h, and then centrifuged at 4°C for 30 min at 13,500 rpm. The supernatant containing the protein extract was collected and an aliquot was subjected to Bio-Rad protein assay. A similar protein extraction protocol in *Xenopus* oocytes was described previously (Bali et al., 2009).

### Immunoblotting

Equal amounts of soluble proteins (unless noted otherwise) extracted from oocytes were resolved by SDS-PAGE under non-reducing conditions and electrotransferred to a polyvinylidene fluoride membrane in a buffer containing 25 mM Tris, 192 mM glycine, 0.1% (w/v) SDS, and 20% (v/v) methanol for 1 h at 18 V. Membranes were briefly rinsed in a buffer containing 10 mM Tris HCl (pH 8.0), 150 mM NaCl, and 0.01% (v/v) Tween-20 (TBS-T) and then incubated at room temperature for 1 h in TBS-T containing 5% (w/v) skimmed milk powder to block non-specific binding of antibodies. Incubation of membranes with rabbit polyclonal antibody to GlyR alpha 1 (catalog number ab475, Abcam, Cambridge, England) was performed in TBS-T containing 1% skimmed milk and 1% BSA powders (w/v) for 2 h at room temperature, followed by three 10-min washes with TBS-T alone. Membranes were then incubated for 1 h at room temperature with an appropriate secondary antibody conjugated to horseradish peroxidase (HRP), diluted in TBS-T containing 1% skimmed milk and 1% BSA powders, followed by three 5-min washes with TBS-T. After the final wash, blots were immediately developed by applying the Enhanced Chemi-Luminescence (ECL) reagent (Pierce Chemical Co., Rockford, IL) for 2 min, and then a Kodak Image Station 2000 MM (Eastman Kodak, Rochester, NY) was used to acquire images.

### Protein identification

For mass spectrometry experiments, 60 μg of protein obtained from oocytes for each condition were resolved by 12% SDS-PAGE under non-reducing conditions and the resulting gels were subjected to Coomassie staining (Imperial Protein Stain, Thermo Fisher Scientific, Waltham, MA). Discrete specific gel bands of interest ranging from ~30 to ~140 kDa were excised, washed 3 times with ultrapure water, and digested in-gel with modified porcine trypsin protease (Promega, Fitchburg, WI). The digested tryptic peptides were desalted using a Zip-tip C18 (Millipore, Billerica, MA). Peptides were eluted from the Zip-tip with 0.5 μl of Matrix Solution (α-cyano-4-hydroxycinnamic acid, 5 mg/ml in 50% acetonitrile, 0.1% trifluoroacetic acid, and 25 mM ammonium bicarbonate) and spotted on a MALDI plate.

#### Mass spectrometry

MALDI-ToF MS and ToF/ToF tandem MS/MS were performed on an AB SCIEX ToF/ToF™ 5800 System (AB SCIEX, Framingham, MA). MALDI-ToF mass spectra were acquired in reflectron positive ion mode, averaging 4000 laser shots per spectrum. ToF/ToF tandem MS fragmentation spectra were acquired for each sample, averaging 4000 laser shots per fragmentation spectrum on each of the 7–10 most abundant ions present in each sample (excluding trypsin autolytic peptides and other known background ions). Protein identification by MS was performed by Applied Biomics (Hayward, CA).

#### Database search

The resulting peptide mass and the associated fragmentation spectra were submitted to GPS Explorer workstation equipped with MASCOT search engine (Matrix Science, Boston, MA) to search the Swiss-Prot database. Searches were performed without constraining protein molecular weight or isoelectric point, with variable carbamidomethylation of cysteine and oxidation of methionine residues, and with one missed cleavage allowed in the search parameters. Proteins with a protein score C.I. % or total ion C.I. % greater than 90 are considered confidence hits. When the protein score C.I. % was lower than 90, confidence was assumed on the basis of different species background and complementary immunoblot evidence.

### Quantification of band ratios

ImageJ64 software (National Institutes of Health, Bethesda, MD) was used to process and quantify GLRA1-labeled band intensity at approximately 50 and 100 kDa, which correspond to the loci at which monomeric and dimeric GlyR subunits, respectively, were identified by MS. These band intensities were then calculated and reported as direct ratios (100:50 kDa), and statistically significant differences in band ratios between the uncrosslinked and crosslinked conditions were measured for the representative TM1-3 mutant, TM3-4 mutant, and wild-type using *t*-tests (*P* < 0.05).

### Molecular modeling

A homology model of homo-pentameric GlyR alpha 1 subunits was built by threading the primary sequence onto the X-ray structure of the eukaryotic GluCl (PDB ID 3RHW) (Hibbs and Gouaux, 2011). The cytoplasmic TM3-4 loop was trimmed to match the length of the GluCl template in that region (Bertaccini and Trudell, 2002). The modeling and subsequent refinement were essentially as described previously (Bertaccini et al., 2005) using the Discovery Studio software suite (Biovia, San Diego, CA) (McCracken et al., 2010).

## Results

### Effect of TM1-TM3 inter-subunit crosslinking on alcohol and anesthetic modulation

**Figure 1:**
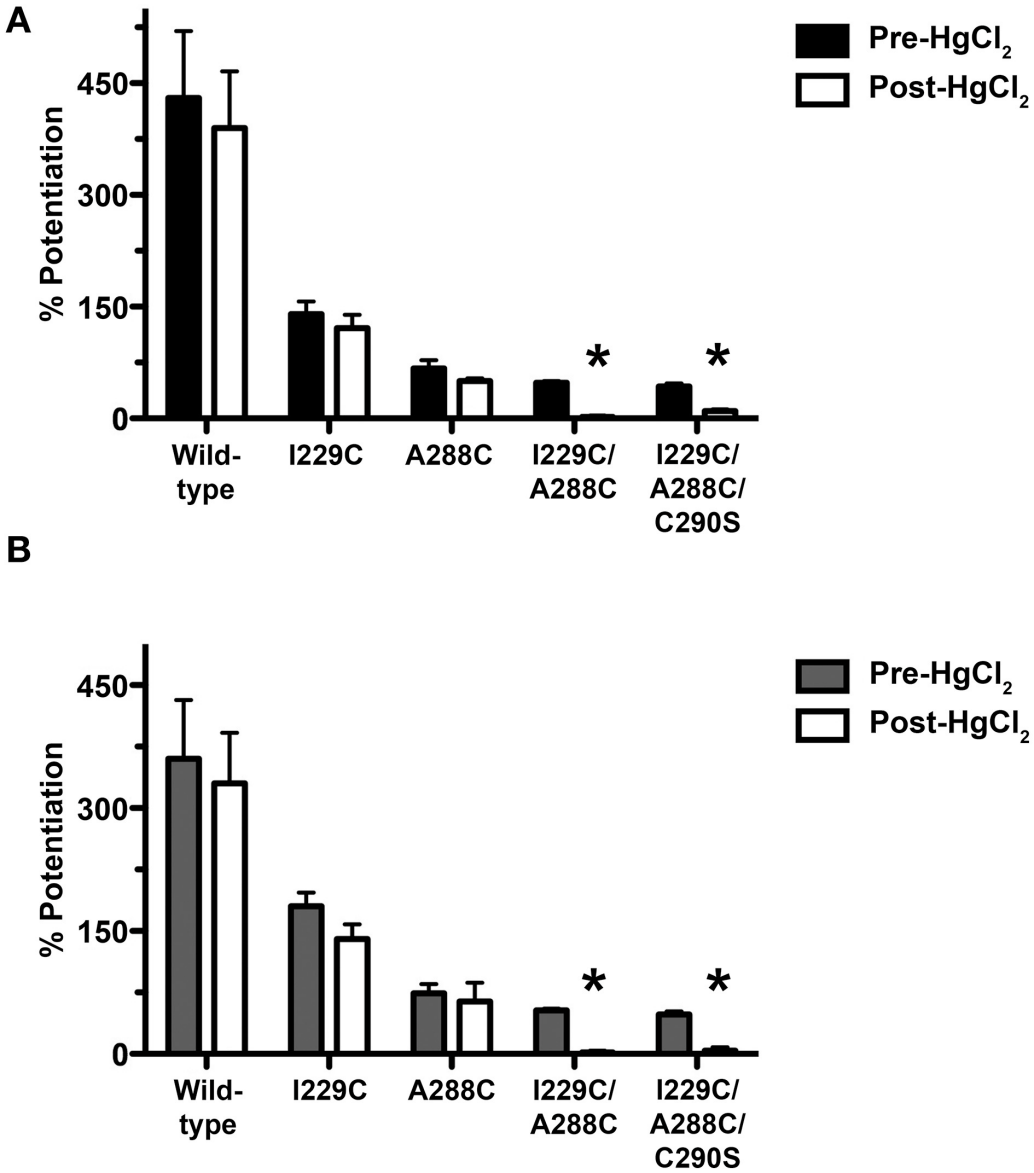
**Effect of crosslinking on alcohol/anesthetic responses in TM1-3 mutants**. Potentiation of EC_5−10_ glycine responses by 22 mM butanol **(A)** or 0.6 mM isoflurane **(B)** before and after application of the crosslinking agent HgCl_2_. HgCl_2_ (10 μM) was applied to wild-type, single mutants, and I229C/A288C and I229C/A288C/C290S mutants in the presence of 100 mM glycine (i.e., the activated/desensitized state). Values represent mean ± SEM from 4 to 5 oocytes. Repeated measures *t*-tests were used to test differences between pre-and post-HgCl_2_ conditions (^*^P < 0.05 compared to the pre-HgCl_2_ condition for each receptor).

We determined if crosslinking of accessible residues A288 in TM3 and I229 in TM1 alters alcohol or volatile anesthetic enhancement of GlyR function in *Xenopus* oocytes. The I229C/A288C double and I229C/A288C/C290S triple mutants and corresponding single mutants were each expressed in oocytes. We prepared the triple mutation in order to rule out possible crosslinking with the native wild-type C290. The mutants form functional homomeric channels, although the GlyR sensitivity differs for some of the mutants (Lobo et al., 2008). We measured the potentiation of submaximal (EC_5−10_) glycine-induced current by 22 mM butanol before and after application of the crosslinking agent HgCl_2_. As previously reported (Lobo et al., 2008), agonist-induced rotation within the TM domain appears to be necessary for the formation of crosslinks in the I229C/A288C mutant; thus HgCl_2_ (10 μM) was applied in the presence of a maximal concentration of glycine (100 mM). HgCl_2_had no effect on butanol potentiation in the wild-type or single mutants, but nearly abolished the potentiation in the double and triple mutants (Figure 1A).

We then measured the potentiation of submaximal (EC_5−10_) glycine-induced current by 0.6 mM isoflurane before and after application of HgCl_2_. As described above for butanol, HgCl_2_ was applied in the presence of 100 mM glycine. HgCl_2_ had no effect on isoflurane potentiation in wild-type or single mutants, but nearly eliminated the potentiation in the I229C/A288C double and I229C/A288C/C290S triple mutants (Figure 1B).

### Effect of TM3-4 intra-subunit crosslinking on alcohol and anesthetic modulation

We determined if crosslinking of A288 in TM3 with critical TM4 residues alters alcohol or volatile anesthetic enhancement of GlyR function in oocytes. The four double cysteine mutants (A288C/Y406C, A288C/W407C, A288C/I409C, and A288C/Y410C), previously shown to form crosslinks (McCracken et al., 2010), and the corresponding single mutants were each expressed in oocytes. The mutants formed functional channels, although the mutations can produce altered glycine sensitivity (Lobo et al., 2006; McCracken et al., 2010).

**Figure 2:**
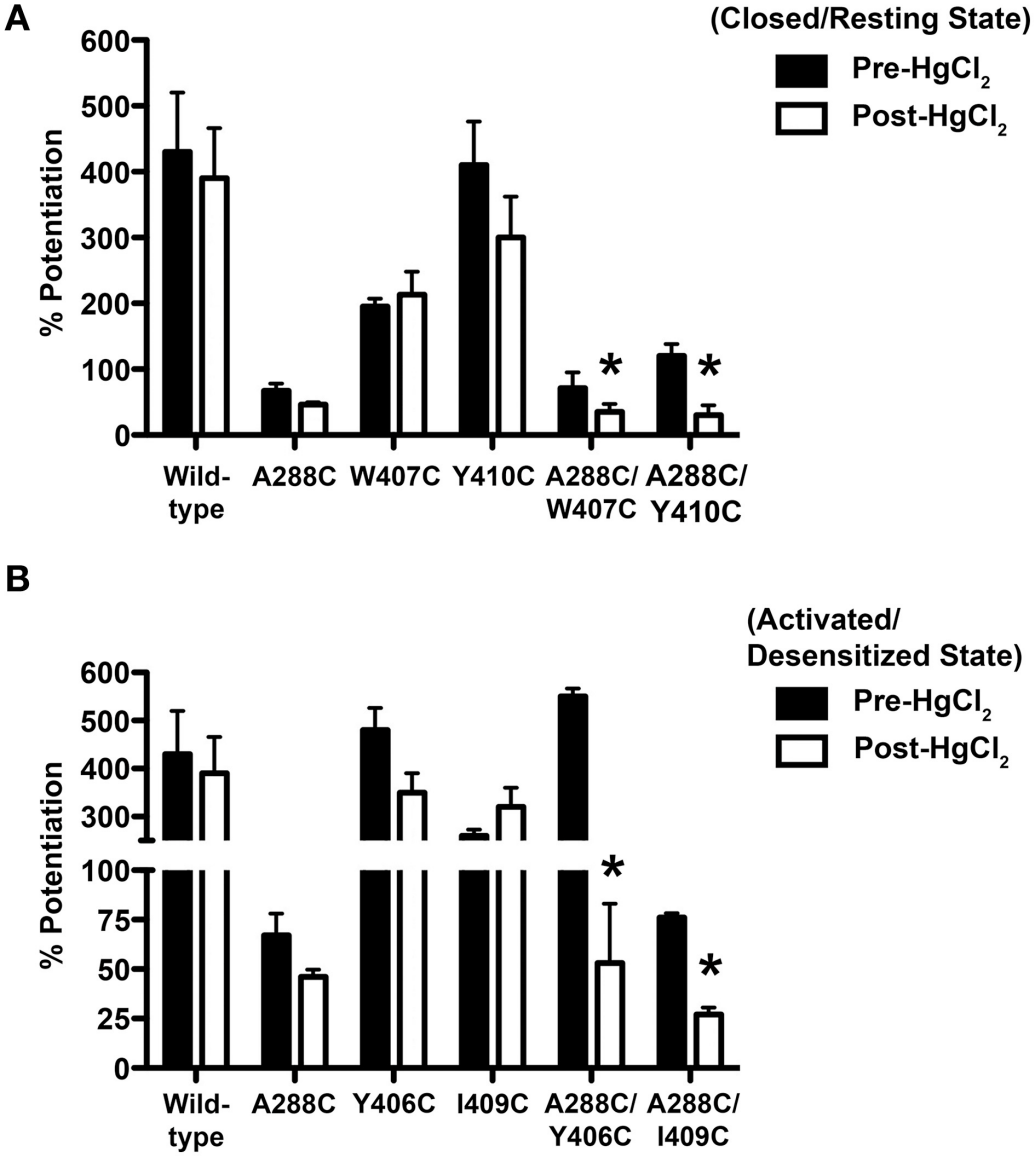
**Effect of crosslinking on alcohol responses in TM3-4 mutants**. Potentiation of EC_5−10_ glycine responses by 22 mM butanol before and after HgCl_2_. **(A)** HgCl_2_ (10 μM) was applied to wild-type, single mutants, and double mutants (A288C/W407C, A288C/Y410C) in the absence of glycine (i.e., the resting/closed state). **(B)** HgCl_2_ (10 μM) was applied to wild-type, single mutants, and double mutants (A288C/Y406C, A288C/I409C) in the presence of 100 mM glycine (activated/desensitized state). Values represent mean ± SEM from 4 to 5 oocytes. Data from wild-type and A288C receptors were combined across experiments and are represented in **(A,B)** and Figure 1A. Repeated measures *t*-tests were used to detect differences between the pre-and post-HgCl_2_ conditions (^*^P < 0.05 compared to the pre-HgCl_2_ condition for each receptor).

**Figure 3:**
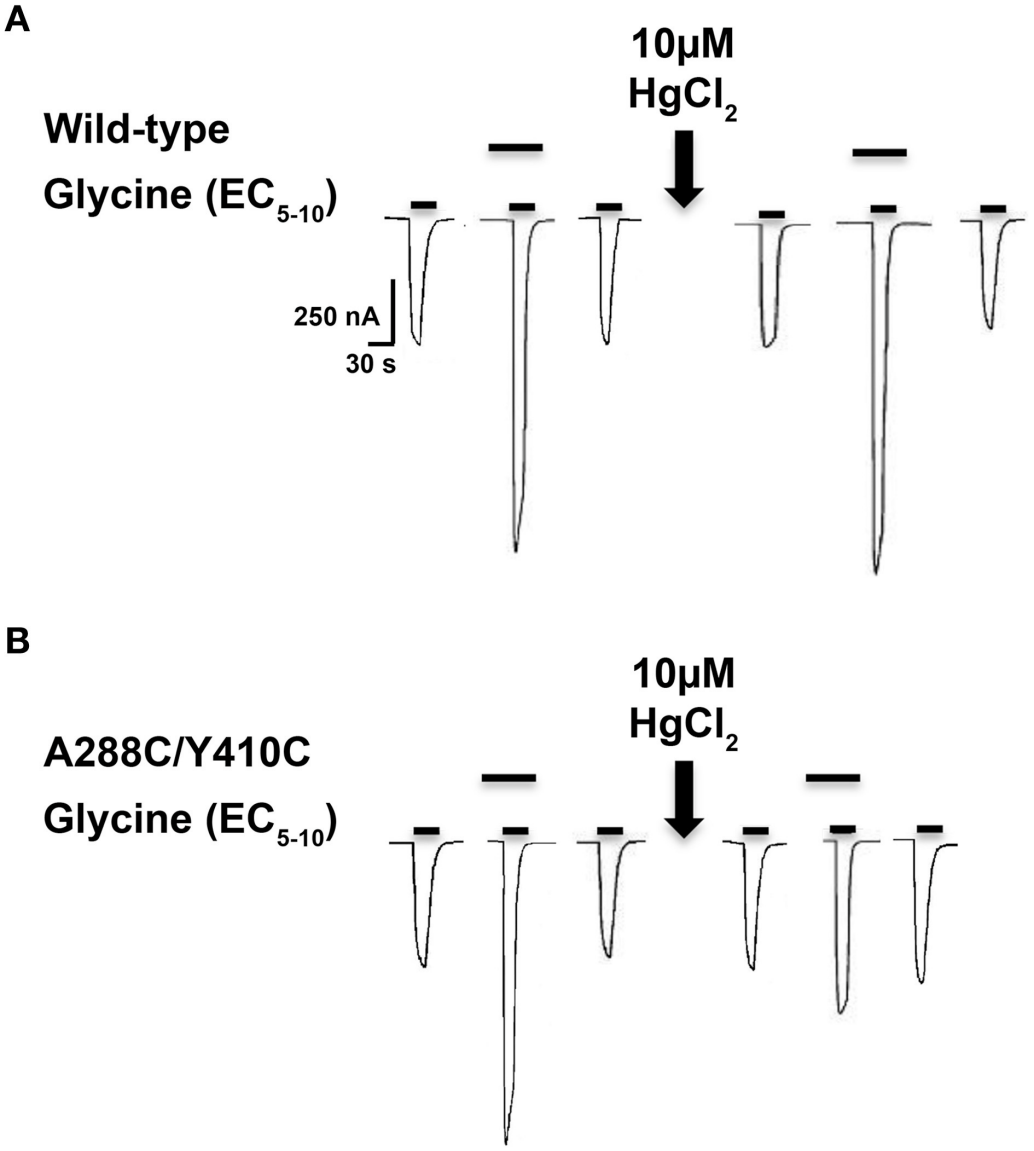
**Effect of crosslinking on alcohol responses in TM3-4 mutants**. Tracings showing the effect of 22 mM butanol before and after application of 10 μM HgCl_2_ on EC_5−10_ glycine-induced currents in individual oocytes expressing wild-type **(A)** or A288C/Y410C **(B)** GlyRs. The upper bars indicate butanol pre-application.

As previously described (McCracken et al., 2010), 10 μM HgCl_2_ was applied to the A288C/W407C and A288C/Y410C double mutants and corresponding single mutants in the absence of glycine (i.e., in the closed/resting state; Figure 2A). However, agonist-induced rearrangement of the TM domain is necessary for the formation of crosslinks in A288C/Y406C and A288C/I409C; therefore HgCl_2_ was applied in the presence of a maximal glycine concentration (i.e., in the open/desensitized state) for these double and corresponding single mutants (Figure 2B). HgCl_2_ had no effect on 22 mM butanol potentiation of glycine responses in wild-type or single mutants, but decreased butanol potentiation in all four double cysteine mutants (Figures 2A,B). Figure 3 shows representative tracings of the butanol effects before and after crosslinkingon glycine-induced currents in individual oocytes expressing wild-type or A288C/Y410C GlyRs.

**Figure 4:**
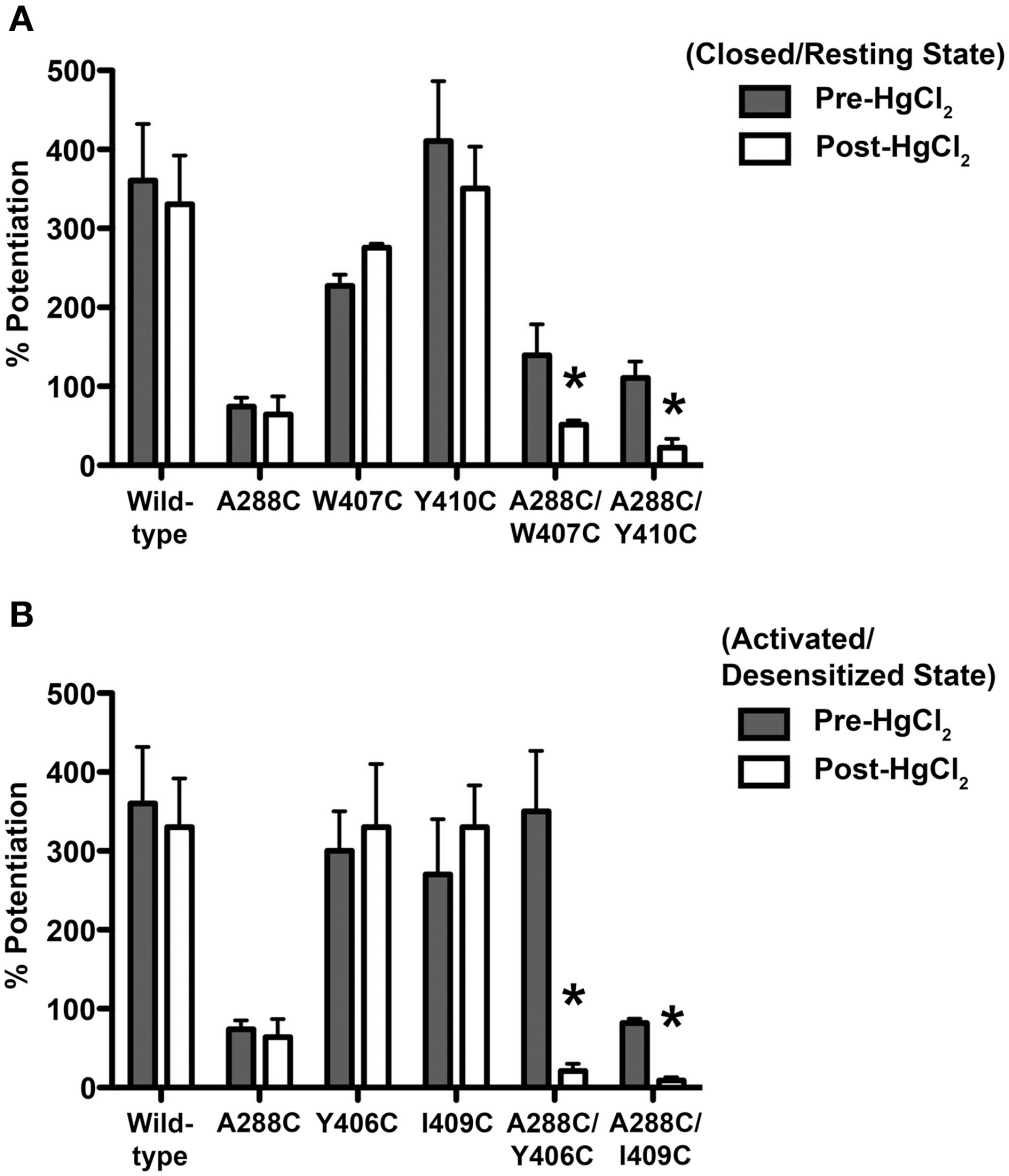
**Effect of crosslinking on anesthetic responses in TM3-4 mutants**. Potentiation of EC_5−10_ glycine responses by 0.6 mM isoflurane before and after HgCl_2_. **(A)** HgCl_2_ (10 μM) was applied to wild-type, double (A288C/W407C, A288C/Y410C), and single mutants in the absence of glycine (closed state). **(B)** HgCl_2_ (10 μM) was applied to wild-type, double (A288C/Y406C, A288C/I409C), and single mutants in the presence of 100 mM glycine (activated/desensitized state). Values represent mean ± SEM from 4 to 5 oocytes. Data from wild-type and A288C receptors were combined across experiments and are represented in **(A,B)** and Figure 1B. Repeated measures *t*-tests were used to detect differences between the pre-and post-HgCl_2_ conditions (^*^P < 0.05 compared to the pre-HgCl_2_ condition for each receptor).

Next we compared the potentiation of EC_5−10_ glycine currents by 0.6 mM isoflurane before and after HgCl_2_ applied during the closed or open channel conditions described previously for each mutant. HgCl_2_ had no effect on wild-type or single mutants, but potently inhibited isoflurane potentiation in the double cysteine TM3-4 mutants (Figures 4A,B).

We addressed the possibility that the diminished butanol/isoflurane effects in the TM3-4 mutants after HgCl_2_ might be due to Hg^2+^ binding to each of the substituted cysteines and forming S-Hg-Cl bonds, rather than forming a disulfide crosslink between TM3 and TM4. Such covalent attachment of a single HgCl could interfere with the ability of butanol/isoflurane to bind or produce an effect at a TM domain cavity. It is unlikely that formation of S-Hg-Cl bonds could explain the reduced butanol/isoflurane effects because the effects were not altered in the single mutants. Nevertheless, we measured butanol and isoflurane modulation in the A288C/Y406C and A288C/I409C mutants when HgCl_2_ was applied in the absence of glycine, which likely prevents alcohol/anesthetic effects by preventing crosslinking as reported previously (McCracken et al., 2010). Potentiation of EC_5−10_ glycine responses by 22 mM butanol or 0.6 mM isoflurane was not altered in the A288C/Y406C mutant under crosslinking conditions in the absence of glycine (data not shown). HgCl_2_ (in the absence of glycine) also had no effect on butanol or isoflurane modulation of the A288C/I409C mutant (data not shown).

### Crosslinking using H_2_O_2_

**Figure 5:**
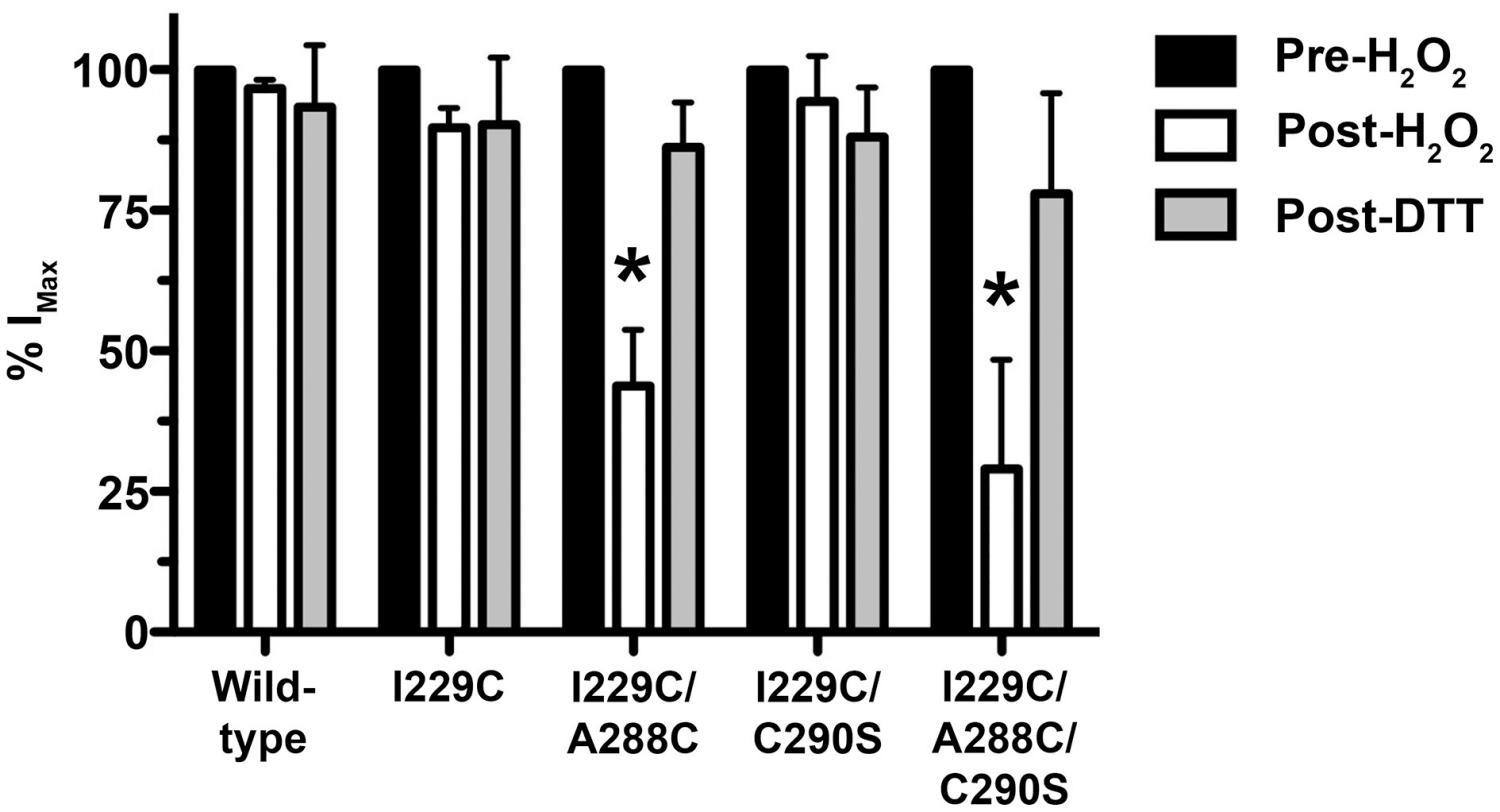
**Effect of crosslinking using H_**2**_O_**2**_ on maximal glycine responses in TM1-3 mutants**. The oxidizing agent, H_2_O_2_ (0.5%), was applied in the presence of 100 mM glycine for 1 minute followed by a 15-min washout. The response to glycine was measured and then the reducing agent dithiothreitol (DTT, 10 mM) was applied for 3 min followed by a 15-min washout. Values represent mean ± SEM from 3 to 6 oocytes (one-way ANOVA with Tukey's *post-hoc* test, ^*^P < 0.05).

The A288C/Y410C mutant showed evidence of crosslink formation using either HgCl_2_ (S-Hg-S) or the oxidizing agent H_2_O_2_ to induce disulfide bonds (S-S) (McCracken et al., 2010). We tested the effectiveness of H_2_O_2_ in the I229C/A288C mutant in order to verify crosslinking by an alternative method in preparation for the proteomic experiments described below. We observed evidence for crosslinking in the I229C/A288C mutant after H_2_O_2_ similar to what we had previously observed using HgCl_2_. We also tested the I229C/C290S and I229C/A288C/C290S mutants to address the possibility that I229C in TM1 may crosslink with the endogenous cysteine at position 290, instead of position A288C. In agreement with Lobo et al. (2008), crosslinking was observed for the I229C/A288C double and I229C/A288C/C290S triple mutants following application of 0.5% H_2_O_2_, based on the significantly decreased glycine-induced current and subsequent reversal by disulfide bond reduction using 10 mM DTT (Figure 5). However, no crosslinking was observed for the wild-type, I229C single mutant, or I229C/C290S double mutant (Figure 5). The I229C/A288C/C290S triple mutant appears to form crosslinks in an almost identical manner to that of I229C/A288C, suggesting that the endogenous cysteine at position 290 is not responsible for the observed effects and that crosslinking is likely occurring between I229 in TM1 and A288 in TM3 of the adjacent subunit. Therefore, the I229C/A288C/C290S mutant was not included in the remainder of the experiments, and we focused on I229C/A288C as the representative TM1-3 mutant for further study.

### Protein identification

The presence of GlyR proteins expressed in oocytes injected with wild-type or representative TM1-3 double mutant (I229C/A288C) was verified by mass spectrometry under uncrosslinked and crosslinked conditions. Proteins were resolved by 12% SDS-PAGE and 26 discrete specific bands ranging from ~30 to ~140 kDa were visualized by Coomassie staining, excised, digested with trypsin, and subjected to MALDI ToF/ToF MS analysis (Table 1). Although the majority of the proteins identified from the MS data originated from *Xenopus laevis*, peptides matching GLRA1 sequence were detected in 16 bands by the MASCOT search engine (Table 2). Relative protein C.I.% scores were slightly below the 90% confidence interval (Supplementary Data); nevertheless, we considered them as correct IDs considering the low amount of human GLRA1 (compared to oocyte-derived proteins) and because the presence of GLRA1 proteins in the preparation was validated by immunoblotting. Among the proteins expressed by oocytes injected with wild-type GlyRs, GLRA1 matching peptides were only identified in the 50 kDa gel bands and not in the ~100 kDa bands, under uncrosslinked and crosslinked conditions. In the case of double-mutants, GLRA1 peptides were identified in bands of a broader molecular weight range; however, protein C.I.% scores relative to the ~100 kDa bands were higher under crosslinking conditions. Further inspection of the matching peptide sequences (Table 2) revealed coverage for several GlyR subdomains, including the Cys-loop region. Thus, MS analysis provided evidence for expression of both monomeric and dimeric GlyR alpha 1 subunits in oocytes.

**Table 1:** **GLRA1 protein identification by mass spectrometry**.

**#**	**Sample type**	**H_2_O_2_**	**Gel band MW (kDa)**	**Peptide count**	GLRA1 matching peptides^*^	**Protein score**	**Protein score C.I.%**
1	WT	No	115–120		–		
2			100–105		–		
3			100		–		
4			50–55	3	*d^*O*^, d^*CO*^, j^*O*^, m^*O*^*	7	60
5	WT	Yes	115–120		–		
6			100–105		–		
7			100		–		
8			50–55	3	*d^*O*^, j^*O*^, m^*O*^*	7	59
9	DM	No	130–140		–		
10			115–120	3	*g, h^*C*^, j^*O*^*	7	59
11			100–105	3	*g, h^*C*^, j^*O*^*	7	59
12			100		–		
13			50–55	4	*f, h^*C*^, j^*O*^, m^*O*^*	10	78
14			48–50	4	*b, h^*C*^, j^*O*^, l*	10	78
15			35	3	*f, g, j^*O*^*	7	60
16			30	4	*c, g, h^*C*^, j^*O*^*	9	76
17	DM	Yes	130–140		–		
18			115–120	3	*e, g, h^*C*^*	7	57
19			100–105	4	*g, h^*C*^, j^*O*^, m*	10	78
20			100	3	*g, h^*C*^, j^*O*^*	7	59
21			50–55	5	*a^*O*^, f, h^*C*^, j^*O*^, m^*O*^*	12	87
22			48–50	4	*b, h^*C*^, j^*O*^, l*	10	78
23			40	4	*g^*C*^, h^*C*^, j^*O*^, k*	9	77
24			35–37		–		
25			30–32	5	*c, g, h^*C*^, i, j^*O*^*	12	89
26			28–30	5	*c, g, h^*C*^, i, j^*O*^*	13	89

*For mass spectrometry experiments, proteins expressed in oocytes injected with wild-type (WT) or double mutant (DM), under uncrosslinked and crosslinked conditions, were resolved by 12% SDS-PAGE and the resulting gels were subjected to Coomassie staining. Twenty-six discrete bands ranging from ~30 to ~140 kDa were excised, digested with trypsin, and subjected to MALDI-ToF MS. GLRA1 theoretical molecular weight is 52,589.7 Daltons and the pI is 8.97. Apparent molecular weights of gel bands are indicated. GLRA1 was detected in 16 bands, with several matching peptides. Protein score C.I.% indicates the confidence of the protein ID calculated from MS data. Note that the number of peptides sequenced does not directly correlate to quantity of protein*.

* *For information on matching peptide sequences, see Table 2*.

**Table 2:** **GLRA1 matching peptide sequences identified by mass spectrometry**.

>sp|P23415|GLRA1_HUMAN			
MYSFNTLRLY LWETIVFFSL AASKEAEAAR SAPKPMSPSD FLDKLMGRTS GYDARIRPNF 60			
KGPPVNVSCN IFINSFGSIA ETTMDYRVNI FLRQQWNDPR LAYNEYPDDS LDLDPSMLDS 120			
IWKPDLFFAN EKGAHFHEIT TDNKLLRISR NGNVLYSIRI TLTLACPMDL KNFPMDVQTC 180			
IMQLESFGYT MNDLIFEWQE QGAVQVADGL TLPQFILKEE KDLRYCTKHY NTGKFTCIEA 240			
RFHLERQMGY YLIQMY**I**PSL LIVILSWISF WINMDAAPAR VGLGITTVLT MTTQSSGSRA 300			
SLPKVSYVKA IDIWM**A**VCLL FVFSALLEYA AVNFVSRQHK ELLRFRRKRR HHKSPMLNLF 360			
QEDEAGEGRF NFSAYGMGPA CLQAKDGISV KGANNSNTTN PPPAPSKSPEEMRKLFIQRA 420			
KKIDKISRIG FPMAFLIFNM FYWII**Y**KIVR REDVHNQ			
**#**	**Start–End**	**Domain**	**Peptide Sequence and PTM**
*a^*O*^*	31–44	Extracellular loop	SAPKPMSPSDFLDK
*b*	94–100	Extracellular loop	QQWNDPR
*c*	133–144	Extracellular loop	GAHFHEITTDNK
*d^*O*^*	151–171	Extracellular loop	NGNVLYSIRITLTLACPMDLK
*d^*CO*^*	151–171	Extracellular loop	NGNVLYSIRITLTLACPMDLK
*e*	160–171	Extracellular loop	ITLTLACPMDLK
*f*	225–234	Extracellular loop, Cys-Loop	YCTKHYNTGK
*f^*C*^*	225–234	Extracellular loop, Cys-Loop	YCTKHYNTGK
*g*	229–241	Extracellular loop	HYNTGKFTCIEAR
*g^*C*^*	229–241	Extracellular loop	HYNTGKFTCIEAR
*h^*C*^*	235–246	Extracellular loop, Cys-Loop	FTCIEARFHLER
*i*	281–299	TM2	VGLGITTVLTMTTQSSGSR
*j^*O*^*	351–369	Cytoplasmic loop	HHKSPMLNLFQEDEAGEGR
*k*	370–385	Cytoplasmic loop	FNFSAYGMGPACLQAK
*l*	392–407	Cytoplasmic loop	GANNSNTTNPPPAPSK
*m*	408–414	Cytoplasmic loop	SPEEMRK
*m^*O*^*	408–414	Cytoplasmic loop	SPEEMRK

*Human GLRA1 protein sequence in FASTA format. Matching peptides identified by mass spectrometry experiments are highlighted. Note that the numbering for all amino acid positions cited in the text is relative to a shorter sequence lacking the first 28 amino acids, for consistency with previous homology and modeling studies. Hence I229, S267, and A288 correspond to I257, S295, and A316 in the FASTA sequence; Y410 corresponds to Y446. These four amino acids are in bold text. In the columns, peptides matching GLRA1 sequence identified with the MASCOT search engine following mass spectrometry. Sequences with amino acid residues, locations, and post translational modifications (PTM) of peptides are indicated. Specific information on peptide sequence matches for each gel band is included in Table 1. Superscripts O and C denote Oxidation and Carbamidomethylation, respectively, in the highlighted amino acid*.

### Immunoblotting

**Figure 6:**
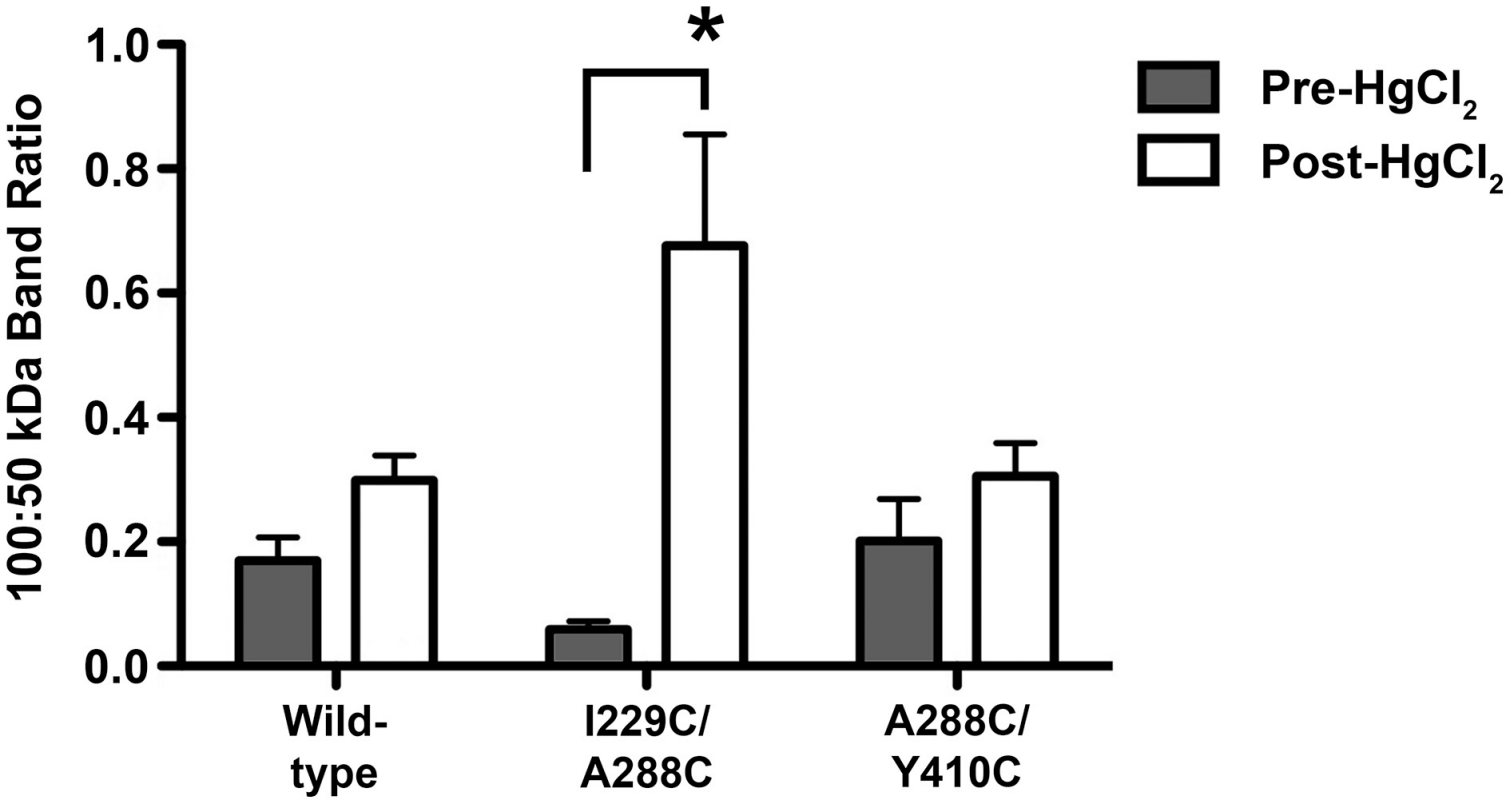
**Semiquantitative analysis of dimeric/monomeric band ratios resulting from uncrosslinked and crosslinked GlyRs**. Crosslinking was obtained by applying 0.5% H_2_O_2_ by bath perfusion. Equal amounts of protein were extracted from oocytes, resolved by SDS-PAGE under non-reducing conditions, and transferred to a membrane where a GlyR alpha 1 antibody was used. Immunoblot images were processed using ImageJ64 software, and GLRA1-labeled band intensity was obtained for 50 and 100 kDa bands, which correspond to monomeric and dimeric GlyR subunits, respectively. Band intensities are reported as direct ratios (100:50 kDa). Immunoblot image analysis revealed a significant increase in the 100:50 kDa band ratio for the I229/A288C (TM1-3) double mutant after crosslinking, but not in the uncrosslinked mutant or in the corresponding wild-type samples. Conversely, no differences in the 100:50 kDa band ratios were detected between uncrosslinked (pre-H_2_O_2_) and crosslinked (post-H_2_O_2_) conditions for the A288C/Y410C (TM3-4) double mutant or in the corresponding wild-type samples. Statistically significant differences in band ratios between the pre-H_2_O_2_ and post-H_2_O_2_ conditions were measured and reported as a ratio (mean ± SEM; *t*-tests, ^*^P < 0.05).

To further investigate whether the proposed crosslinks are formed between residues within the same subunit (intra-subunit) or between subunits (inter-subunit), and whether these residues are participating in alcohol sites of action within or between GlyR subunits, we extracted proteins from oocytes injected with wild-type, TM3-4 mutant (A288C/Y410C), or TM1-3 mutant (I229C/A288C) GlyRs. We then used immunoblotting with a GlyR alpha 1 antibody. When electrophoretically separated, along with the proteins extracted from wild-type injected oocytes, expressed GlyRs were clearly detectable by immunoblotting as a distinct ~50–52 kDa band, as shown in Supplementary Figure 1. If crosslinking occurs between two adjacent subunits, an increase in the presence of a GlyR-labeled ~100 kDa band (accompanied by a relative increase in the 100:50 kDa band ratio) is expected under non-reducing conditions. Conversely, no change would be expected in this ratio if crosslinking occurs within a single subunit. Thus, the differences in the 100:50 kDa band ratios were quantified and compared between uncrosslinked and crosslinked conditions for the representative mutants and wild-type. The band ratio was significantly increased in the I229C/A288C mutant after crosslinking (Supplementary Figure 2 and Figure 6). However, in support of the hypothesis that crosslinks to the TM4 subunit would be intra-subunit, there were no differences in the 100:50 kDa band ratios between the uncrosslinked or crosslinked conditions for the TM3-4 mutant or wild-type (Supplementary Figure 3 and Figure 6).

### GlyR homology modeling

**Figure 7:**
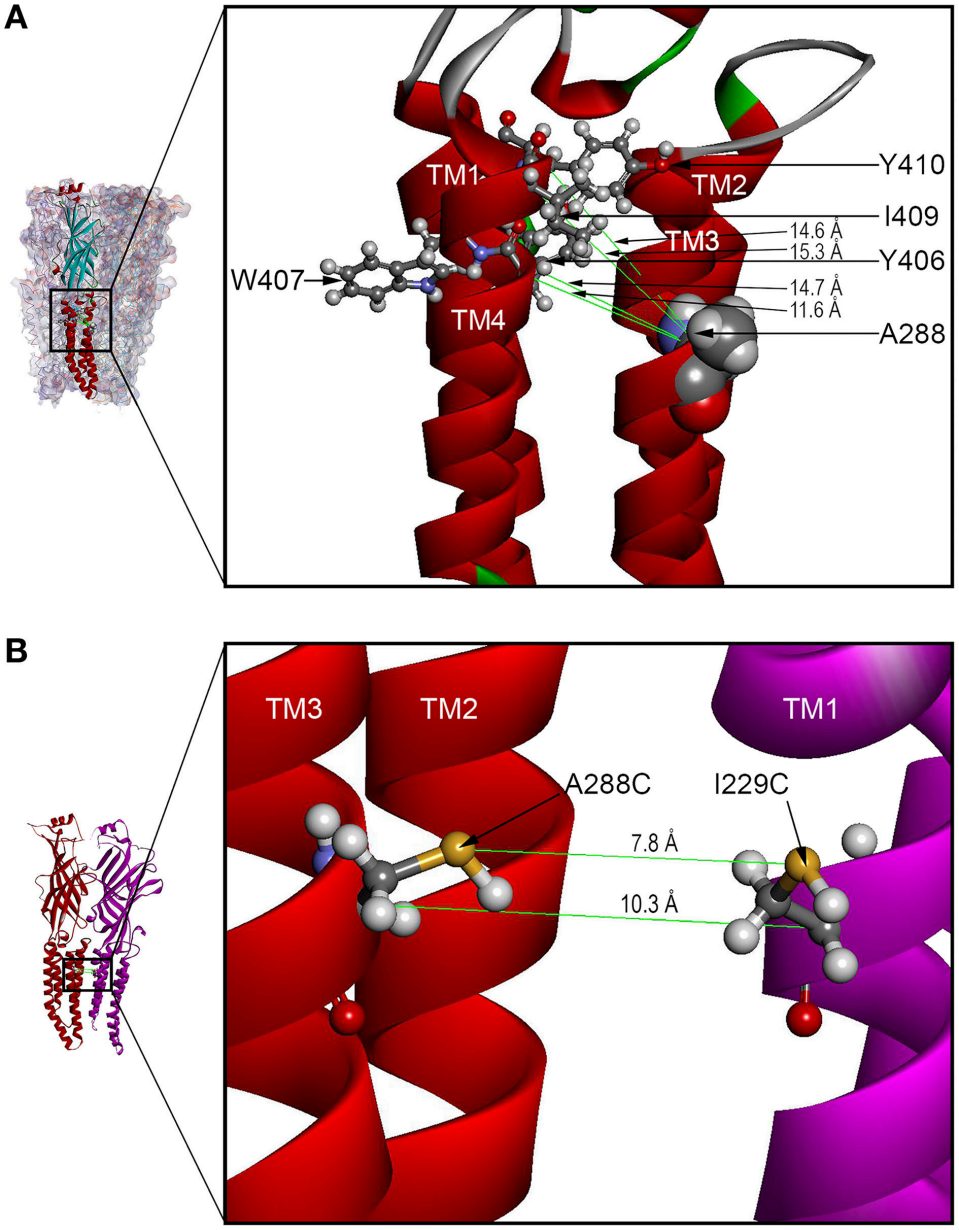
**GLRA1 homology models based on GluCl showing TM3-4 intra-subunit and TM1-3 inter-subunit distances**. **(A)** The enlarged boxed area of a GLRA1 subunit shows distances between the C-alpha atoms of cysteines substituted at A288 (TM3) and Y406, W407, I409, and Y410 (TM4) within the same subunit were 14.7, 11.6, 15.3, and 14.6 Å, respectively. **(B)** The enlarged boxed area of two GLRA1 subunits shows distances between the C-alpha and S-S atoms of adjacent subunits (rendered as backbone ribbons in red and purple) were 10.3 and 7.8 Å, respectively.

Our previous GlyR models were based on the prokaryotic proton-gated GLIC structure (Bocquet et al., 2009). However, in this study the homology model was based on the X-ray structure of the eukaryotic ligand-gated GluCl (PDB ID 3RHW) (Hibbs and Gouaux, 2011). Advantages of using GluCl as a template include: the eukaryotic source, the high resolution of GluCl structural data, and the greater sequence similarity between the GlyR and GluCl compared to GLIC (McCracken et al., 2010). Before using the structure for homology modeling, the antibodies and ivermectin were removed. Unlike the questions raised regarding the alignment (Ernst et al., 2005) of GlyR with nAChR (PDB ID 2BG9) (Unwin, 2005), there were no gaps in the alignment in the TM2-3 loop, and the similarity of residues in TM4 was evident. The refined model was used to suggest mutation of residues of interest to cysteine and measure center-to-center distances between atoms to determine potential sites for crosslinking. Distances between the C-alpha atoms of cysteine substituted at A288 (TM3) and Y406, W407, I409, and Y410 (TM4) within the same subunit were 14.7, 11.6, 15.3, and 14.6 Å, respectively (Figure 7A). In addition, we used both the C-alpha and the S-S bond distances to show the possible inter-subunit distances between the TM3 segment of one subunit and the TM1 subunit of the adjacent subunit; these inter-atomic distances were 10.3 and 7.8 Å, respectively (Figure 7B).

## Discussion

Understanding high-resolution structures of alcohol/anesthetic sensitive ion channels can facilitate the design and future development of improved structure-based drugs (Harris et al., 2008; Heusser et al., 2013; Gorini et al., 2014; Howard et al., 2014). To this end, several studies have explored the biochemical interactions of putative GlyR subunit residues that may participate in alcohol/anesthetic sites of action. Residues A288 in TM3 and Y406, W407, I409, and Y410 in TM4 are water-accessible and may contribute to a hydrophilic, drug pocket (Jenkins et al., 2001; Lobo et al., 2006, 2008; Harris et al., 2008). The proximity of A288 in TM3 to these TM4 residues provided evidence for crosslinking between TM3-4 (McCracken et al., 2010). These studies indicated that TM3-4 regions participate in an intra-subunit cavity. However, the homologous residue to A288 in TM3 of the GABA_A_R is oriented toward the subunit interface such that it is able to form crosslinks with TM1 residues of an adjacent subunit (Bali et al., 2009), and photoaffinity labeling suggested that different classes of general anesthetics may act at an inter-subunit site (Stewart et al., 2008; Zhong et al., 2008; Li et al., 2010; Chiara et al., 2013). Crystallographic studies using an ethanol-sensitized GLIC mutant co-crystallized with ethanol, bromoethanol, and bromoform provided additional support for an inter-subunit drug cavity (Sauguet et al., 2013). These results differ, at least in part, from the recent x-ray structure of desflurane bound at an intra-subunit cavity in the bacterial homolog GLIC (Nury et al., 2010; Howard et al., 2011) and suggest that alcohol and anesthetic interaction with GLIC may involve more than a simple binding cavity operating between or within subunits (Nury et al., 2010).

Based on the studies above, we aimed to determine whether the critical residues are oriented to form a site of interaction within the same subunit, between subunits, or both. We mutated key residues with cysteines, which are able to form disulfide bonds in the presence of crosslinking agents. Then we measured endogenous ligand-mediated current potentiation in the presence of an agonist, assuming that when potentiation occurs, the residue is accessible to the ligand. Conversely, reduced or abolished potentiation after the application of a crosslinking agent implies that the residue is not ligand accessible. A reducing agent (DTT) would break crosslinks and reinstate residue accessibility, as shown in our results. Collectively, our electrophysiological experiments suggest that these residues regulate accessibility to butanol/isoflurane sites of action.

We used mass spectrometry to verify the expression and dimerization of double-mutant GlyR subunits and immunoblotting to compare dimeric/monomeric band ratios in uncrosslinked vs. crosslinked conditions. The reported increase in the 100:50 kDa ratio observed after crosslinking in I229C/288C, but absent in A288C/Y410C mutants or wild-type, provides novel evidence for our model. Indeed, our current work suggests that alcohol and anesthetic interactions with the human GLRA1 subunit involve intra- and inter-subunit sites, depending on the TM regions involved and the channel activation conditions. Although we propose that crosslinking most likely occludes butanol/isoflurane sites and prevent their effects, it is also plausible that drug interaction still occurs, while crosslinking constrains either the side-chain or helix rearrangements that are necessary for drug effects on channel gating.

We initially used the crosslinking agent HgCl_2_, which reacts with accessible cysteine pairs to form intermolecular S-Hg-S dimers when the residues are in proximity to one another and located on opposing faces of adjacent helices (Struthers et al., 2000; Soskine et al., 2002), to provide insight into the orientation and position of TM residues. An ideal C-alpha distance in a disulfide bond is approximately 6 Å. The use of HgCl_2_ to form an S-Hg-S bond extends that bond distance to approximately 8 Å. GlyR homology modeling using the GluCl template showed distances between the C-alpha atoms of cysteine substituted at I229 (TM1), A288 (TM3), and Y406 (TM4) in two adjacent subunits ranged from 7 to 12 Å (Figure 7). Inter-subunit crosslinks between I229 in TM1 and A288 in TM3 would require a substantial degree of flexibility in the upper portion of TM3 and about a 100-degree rotation. This would permit A288 to crosslink with I229 in TM1 across the subunit cleft, or alternatively by rotating in the opposite direction, to crosslink with the residues in TM4 (Y406, W407, I409, Y410) (Figure 7). This seems plausible given that several of these mutants (I229 in TM1 and Y406 and I409 in TM4) require co-application of glycine with the crosslinking agent in order for crosslinking to occur.

The position of A288C in our homology model and in the recent cryo-electron microscopy and X-ray structures of the GlyR (Du et al., 2015; Huang et al., 2015) is facing more toward the inter-subunit space than toward the intra-subunit cavity. However, the homology model shows that the distances between A288C in TM3 of one subunit and residues in TM4 of the adjacent subunit are so great, that in either case, considerable distortion would be required for crosslinking. A more likely possibility is that the crosslinking to TM4 is intra-subunit and the two alpha helices at the extracellular end of the TM domain are flexible. In fact, a recent comparison of propofol binding in the TM domain of the GABA_A_R in the open, closed, and desensitized states revealed that most of the differences between these states occurred at the extracellular end of TM2, the TM2-3 linker, and TM3 (Franks, 2015).

Previously, we considered the effect of a 100-degree rotation of TM3 during the opening transition (McCracken et al., 2010). There are now many reports demonstrating motion of TM3 or global motion within subunits that changes the accessibility of drugs to the intra-subunit cavity (Moraga-Cid et al., 2015). An example of such motion was described in a molecular dynamics simulation of the GluCl-ivermectin structure (Yoluk et al., 2013). A more extreme example is a “spring model” used to explain the effect of substitutions in TM3 of the nAChR (Otero-Cruz et al., 2007). This model proposed extensive “fraying” and extension of the alpha-helical structure at the extracellular end of TM3.

One uncertainty is the position and motion of TM4 in these channels (Bertaccini et al., 2010). High-resolution structures of GLIC, *Erwinia chrysanthemi* ligand-gated ion channel (ELIC), and GluCl find TM4 to be tilted away from the other TM regions (Bocquet et al., 2009; Hilf and Dutzler, 2009; Hibbs and Gouaux, 2011) and the intra-subunit binding site for desflurane in GLIC is formed primarily by TM1-3 (Nury et al., 2010). It is possible that tilting of TM4 allows it to be partially or completely surrounded by lipid (Baenziger and Corringer, 2011). However, TM3 and TM4 were in sufficient proximity to allow intra-subunit crosslinking using either HgCl_2_ or H_2_O_2_ in the A288C/Y410C mutant (McCracken et al., 2010). Other work indicates that W407C, I409C, Y410C, and K411C are accessible to H_2_O_2_ and are likely exposed to water molecules, which is consistent with this region of TM4 contributing to a water-filled cavity (Lobo et al., 2006). It is likely that TM4 has considerable flexibility and may approach the other TM regions, perhaps during gating, which is supported by the observation that crosslinking of A288C/Y406C and A288C/I409C are only observed if the receptor is activated by glycine (McCracken et al., 2010). We propose that alcohols and anesthetics help stabilize the open state of GlyRs because this state facilitates a closer orientation of TM4 to TM1-3, which allows stronger binding to an intra-subunit site formed by all four TM segments (Yoluk et al., 2015).

We note that speculations about “binding sites” or “sites of interaction” are constrained by our studies and those of others (Murail et al., 2011; Yoluk et al., 2015). These studies show that the occupancy time of ligands is very transient (nanoseconds). Thus small ligands, such as butanol and isoflurane, will not reside in a single site during the course of an action potential (microseconds to milliseconds). As a result, we are likely viewing an ensemble average of the interactions of these ligands with GLRA1. Studies of the GABA_A_R showed that under conditions where anesthetics occlude initial rates of substituted cysteine modification, the rate of inhibition (as a measure of anesthetic site occupancy) indicates that a high degree of time-averaged site occupancy can be achieved when receptors are in a high-affinity state or drug concentrations are very high (Stewart et al., 2013).

Our work shows that crosslinking between A288 in TM3 and I229 in TM1 or between A288 and Y406, W407, I409, or Y410 in TM4 strongly reduces the ability of butanol and isoflurane to potentiate glycine-induced currents and supports participation of these residues in alcohol/anesthetic sites of action. The application of different proteomic methodologies provides additional support, suggesting that the TM1 and 3 segments likely participate in an inter-subunit site (between adjacent GlyR alpha 1 subunits), while TM3-4 participate in an intra-subunit site (within the same alpha 1 subunit). The recent publication of a high-resolution structure of a chimera between the TM domain of the GlyR and the extracellular domain of GLIC reveals many details of the interacting domains (Moraga-Cid et al., 2015). Moreover, new electron cryo-microscopy structures of zebrafish GlyRs at intermediate resolutions (Du et al., 2015) provide strong support for our use of the GluCl structure as a template for homology modeling. These new GlyR structures are at an estimated resolution of only 3.8–3.9 Å, and have some missing side chains. In addition, a three-residue loop is substituted for the intracellular TM3-4 loop. Nevertheless, the set of three self-consistent structures in the resting, open, and desensitized states offers many opportunities for their use in directed molecular dynamics simulations of the transitions between these states. Overall, the considerations above support our choice of GluCl as a good template for the present studies.

It is interesting to consider that the protein segments studied here may be part of the dark “proteome” as defined by Perdigão et al. (2015), even though these regions were not considered “dark” under the strict criteria used by Perdigao and coworkers because their structures have been identified in the Protein Data Bank (PDB; Berman et al., 2000). However, inspection of the GlyR in the resting, open, and desensitized states (Du et al., 2015), reveals that these segments spend much of their time undergoing transitions between the three states along unknown pathways. These transition pathways, which include twisting, blooming, and exchange of electrostatic bonds, are supported by our data, which can only be explained by large excursions from the known PDB structures.

Our results are consistent with the x-ray structure of desflurane bound at an intra-subunit cavity in the homolog GLIC (Nury et al., 2010). The high-resolution structure of GLIC with desflurane shows a “linking tunnel” which connects the inter- and intra-subunit cavities (Nury et al., 2010), and alcohol action on mutant GLIC channels suggests that the tunnel may also be important for enhancement of function by short chain alcohols (Howard et al., 2011). This tunnel that links intracellular sites with sites at the domain interface is very similar to a tunnel revealed by previous homology modeling of the GlyR (Crawford et al., 2007). Insights from these structures and our current model using GluCl, combined with our functional evidence in oocytes, point to an interaction of isoflurane and butanol with alpha 1 subunits that is more complex than a single site surrounded by four TM helices. Our results suggest a multi-site model for alcohol/anesthetic interaction within human GLRA1 subunits. In this model, crosslinking may prevent access to sites of drug action or produce conformational changes that interfere with alcohol/anesthetic-induced channel gating.

## Author contributions

Data acquisition (MM, GG, LM); Data analysis and preparation of figures/tables (MM, GG, JT); Study design/interpretation and drafting of manuscript (MM, GG, RM, RH, JT); final approval of manuscript (MM, GG, LM, RM, RH, JT).

## Funding

This work was supported by the National Institutes of Health, National Institute on Alcohol Abuse and Alcoholism (grants R01 AA013378; R37 AA006399; R01 AA020980; U01 AA016648; U01 AA0209260).

### Conflict of interest statement

The authors declare that the research was conducted in the absence of any commercial or financial relationships that could be construed as a potential conflict of interest.
