# Supplementary material for: Inter- and Intra-Subunit Butanol/Isoflurane Sites of Action in the Human Glycine Receptor
Source: Front Mol Neurosci. 2016 Jun 14;9:45. doi: 10.3389/fnmol.2016.00045 (PMC4906044; doi:10.3389/fnmol.2016.00045)

McCracken ML, Gorini G, McCracken LM, Mayfield RD, Harris RA & Trudell JR
Inter- and intra-subunit butanol/isoflurane sites of action in the human glycine receptor
Frontiers in Molecular Neuroscience, 2016
Supplementary Figures

Supplementary Figure 1. Immunoblotting of uncrosslinked wild-type GlyRs. Equal amounts of protein were extracted from oocytes, resolved by SDS-PAGE under non-reducing conditions, transferred to a membrane, and incubated with a GlyR alpha 1 antibody. Immunoblot of 30 and 60 μg total proteins extracted from wild-type injected oocytes revealed a dominant ~50-52 kDa band with increasing concentrations of wild-type GlyR protein; no corresponding bands were detectable in proteins obtained from uninjected oocytes. A faint ~100 kDa band derived from the dimerization of two adjacent GlyR alpha 1 subunits is also detectable in wild-type samples. Antibody heavy chains are visible as ~48-50 kDa bands in the samples obtained from uninjected oocytes.

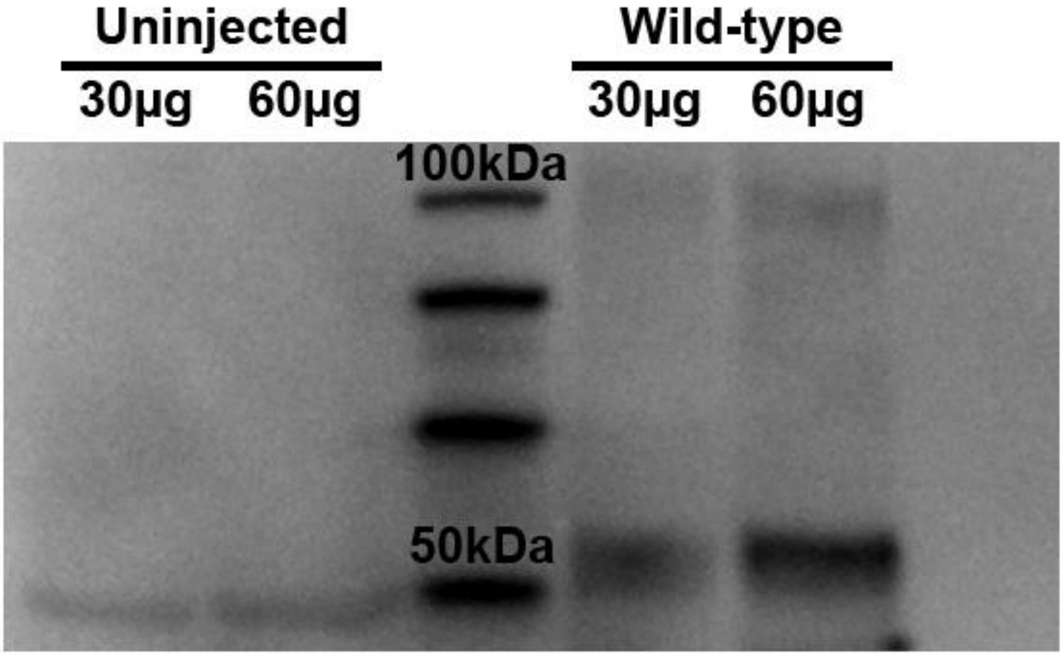

Supplement: Supplementary file 1 [file Image1.PDF]
