# Supplementary material for: Inter- and Intra-Subunit Butanol/Isoflurane Sites of Action in the Human Glycine Receptor
Source: Front Mol Neurosci. 2016 Jun 14;9:45. doi: 10.3389/fnmol.2016.00045 (PMC4906044; doi:10.3389/fnmol.2016.00045)

Supplementary Figure 2. Immunoblotting of uncrosslinked and crosslinked GlyRs in TM1-3 mutants. Crosslinking was obtained by applying 0.5% H_2O_2 by bath perfusion. Equal amounts of protein were extracted from oocytes, resolved by SDS-PAGE under non-reducing conditions, transferred to a membrane, and incubated with a GlyR alpha 1 antibody. A representative immunoblot of proteins extracted from oocytes injected with wild-type or I229C/A288C double mutant *GLRA1* DNA, before and after crosslinking, is shown. Membrane incubation with a GlyR-specific antibody resulted in 50 and 100 kDa distinct bands of different intensity.

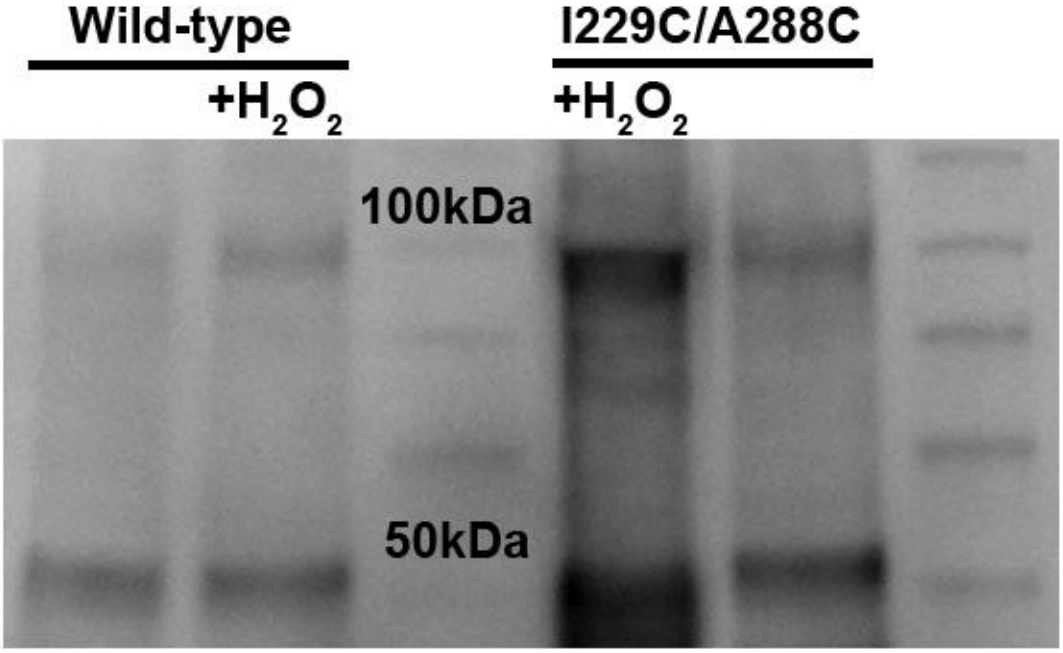

Supplement: Supplementary file 2 [file Image2.PDF]
