# Supplementary material for: Inter- and Intra-Subunit Butanol/Isoflurane Sites of Action in the Human Glycine Receptor
Source: Front Mol Neurosci. 2016 Jun 14;9:45. doi: 10.3389/fnmol.2016.00045 (PMC4906044; doi:10.3389/fnmol.2016.00045)

Supplementary Figure 3. Immunoblotting of uncrosslinked and crosslinked GlyRs in TM3-4 mutants. Crosslinking was obtained by applying 0.5% H_2O_2 by bath perfusion. Equal amounts of protein were extracted from oocytes, resolved by SDS-PAGE under non-reducing conditions, transferred to a membrane, and incubated with a GlyR alpha 1 antibody. A representative immunoblot of proteins extracted from oocytes expressing wild-type or A288C/Y410C double mutant, before and after crosslinking, is shown.

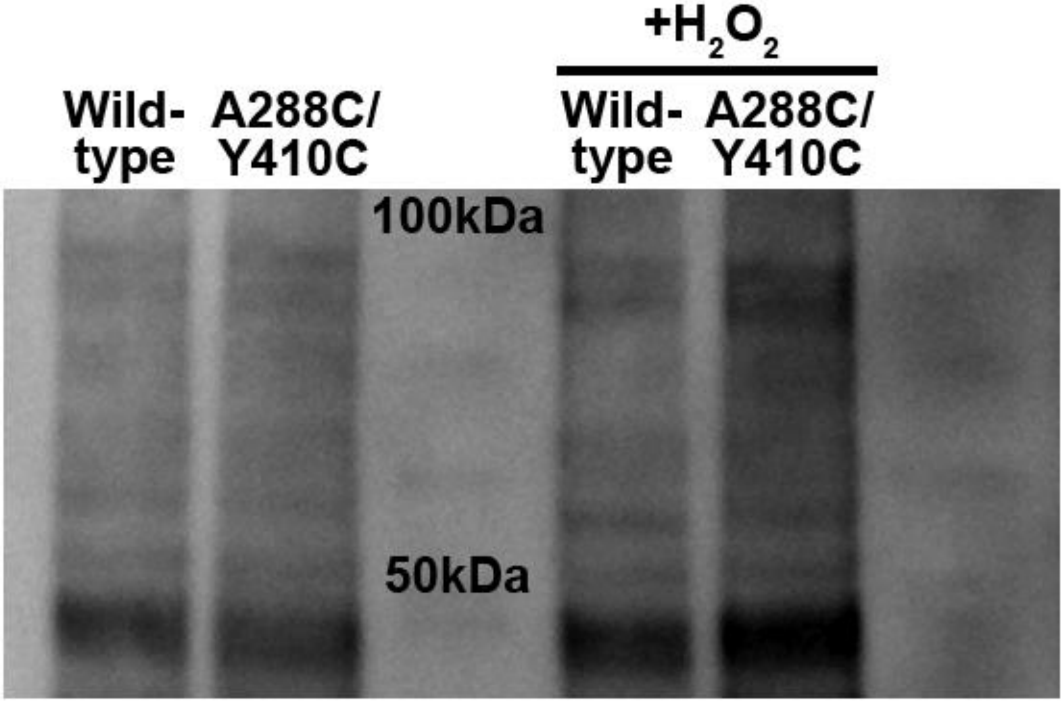

Supplement: Supplementary file 3 [file Image3.PDF]
